# Characterization of MSB Synapses in Dissociated Hippocampal Culture with Simultaneous Pre- and Postsynaptic Live Microscopy

**DOI:** 10.1371/journal.pone.0026478

**Published:** 2011-10-20

**Authors:** James E. Reilly, Hugo H. Hanson, Mónica Fernández-Monreal, Susan L. Wearne, Patrick R. Hof, Greg R. Phillips

**Affiliations:** 1 Department of Neuroscience, Mount Sinai School of Medicine, New York City, New York, United States of America; 2 Computational Neurobiology and Imaging Center, Mount Sinai School of Medicine, New York City, New York, United States of America; Institut de Génomique Fonctionnelle, France

## Abstract

Multisynaptic boutons (MSBs) are presynaptic boutons in contact with multiple postsynaptic partners. Although MSB synapses have been studied with static imaging techniques such as electron microscopy (EM), the dynamics of individual MSB synapses have not been directly evaluated. It is known that the number of MSB synapses increases with synaptogenesis and plasticity but the formation, behavior, and fate of individual MSB synapses remains largely unknown. To address this, we developed a means of live imaging MSB synapses to observe them directly over time. With time lapse confocal microscopy of GFP-filled dendrites in contact with VAMP2-DsRed-labeled boutons, we recorded both MSBs and their contacting spines hourly over 15 or more hours. Our live microscopy showed that, compared to spines contacting single synaptic boutons (SSBs), MSB-contacting spines exhibit elevated dynamic behavior. These results are consistent with the idea that MSBs serve as intermediates in synaptic development and plasticity.

## Introduction

Axonal boutons of excitatory neurons frequently synapse with multiple postsynaptic partners. These have been alternatively termed multisynaptic boutons (MSBs), multisynapse boutons, multiple synapse boutons, and multiple synapses. MSBs generally contact spines containing postsynaptic specializations rather than immature filopodia [1], [2], [3]. Electron microscopy (EM) studies have shown that the number of MSB synapses as a percentage of all synapses can vary from approximately 14% in layer I of the adult mouse neocortex [1] to 19–25% in adult rat hippocampal area CA1 [4], [5]. Some studies have suggested that MSBs most often contact spines from different dendrites [4], [6], while others have shown that both same- and different-dendrite MSB synapses are found in significant numbers [7], [8]. Importantly, primarily same-dendrite MSBs are formed following LTP induction in rat hippocampal slices [8].

Synaptogenic stimuli have been found to be associated with increases in the number of MSB synapses. This has been shown in various paradigms including lesioning [9], [10], [11], [12], [13], hippocampal slice preparation [14], long-term potentiation (LTP) [8], [15], whisker trimming [1], cerebellar motor learning [16], hippocampus-dependent associative learning [17], visual cortex plasticity [18], and estrogen treatment [7], [19]. However, the mechanisms by which MSB synapses are generated, their specific fates, and how they might feature in synaptic plasticity or development are as yet unresolved.

Here, we examined the behavior of MSB-contacting spines using long-term time lapse live microscopy of neurons in dissociated culture with DsRed fluorescently labeled presynaptic boutons contacting GFP fluorescence-filled dendritic spines. Our use of dissociated culture greatly facilitated separate pre- and postsynaptic labeling as it allows each label to be transfected into a separate population of dissociated neurons prior to plating. Obtaining a sufficiently high number of labeled pre-to-postsynaptic contacts among neurons in vivo or in slice culture would be prohibitively difficult. Prior to conducting the live microscopy, we showed that MSB synapses are present at an appreciable level on neurons in dissociated culture. Additionally, we determined that MSB-contacting spines share specific spatial properties distinguishing them from spines contacting single synaptic boutons (SSBs). Finally, our time lapse live microscopy showed that MSB-contacting spines exhibit increased dynamic behavior compared to SSB-contacting spines. These results suggest that, rather than persisting as stable entities, MSB synapses may feature in dynamic processes of neuronal organization.

## Results

### MSBs in dissociated neuron cultures

**Figure 1:**
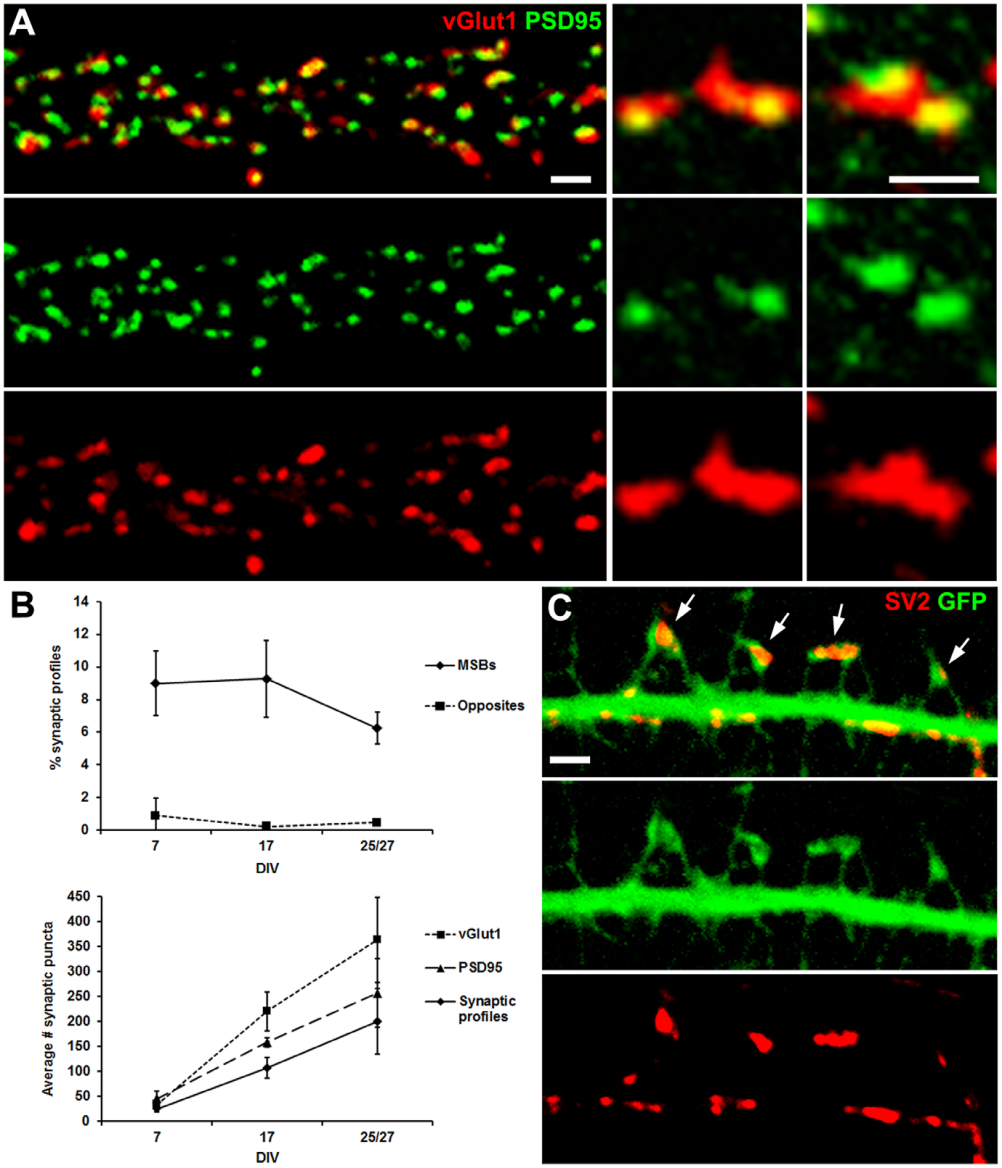
Apparent multisynaptic contacts in immunolabeled cultured neurons. (A, left) LSCM z-stack projection of immunolabeled presynaptic marker vGlut1 (red) and postsynaptic marker PSD95 (green) along a dendritic segment. Scale bar is 2 µm. (A, middle and right) Enlarged examples of vGlut1 puncta in contact with single (middle) and multiple PSD95 puncta (right). Scale bar is 0.5 µm. (B, top) Number of vGlut1 and PSD95 double immunolabeled multiple contacts per neuron as a percentage of all synaptic contacts per neuron with increasing time in culture. Contacts were defined as abutting or overlapping puncta. Fluorescence intensity was normalized across images by adjusting the gain during image acquisition such that the centers of the puncta were at ceiling. Neurons at 25 and 27 DIV were counted together as a single time point. “Opposite” is the number of the synaptic contacts in the arrangement opposite of MSB synapses (PSD95 puncta in contact with multiple vGlut1 puncta) as a percentage of all synaptic profiles. (B, bottom) Average number of double immunolabeled vGlut1 and PSD95 synaptic contacts per neuron with increasing time in culture. Neurons at 25 and 27 DIV were counted together as a single time point. (C) LSCM z-stack projection of a GFP-filled dendritic segment (green) with immunolabeled presynaptic marker SV2 (red). Arrows point to SV2 puncta in contact with multiple GFP-filled spines. Scale bar is 2 µm.

Dissociated neuron cultures provide an optimal system for studying the dynamics of intact synaptic contacts in live neurons because they allow simultaneous visualization of presynaptic boutons in direct contact with their postsynaptic partners, which is prohibitively difficult with slice or *in vivo* preparations. To determine whether MSB synapses are present in dissociated neuron cultures, we performed immunolabeling at various stages of development using antibodies against vGlut1 for presynaptic boutons and PSD95 for postsynaptic specializations (Fig. 1A). Although the majority of vGlut1 presynaptic puncta formed synaptic profiles by contacting single postsynaptic PSD95 puncta (Fig. 1A, middle panels), we observed many in contact with multiple distinct PSD95 postsynaptic puncta (Fig. 1A, right panels), thus fitting the expected profile of an MSB synapse. Quantification of all multiple and single synaptic contacts showed that approximately 8% of the total (158 of 1982) from 7 to 27 DIV fit the MSB synapse profile (Fig. 1B, top). Overall, the average total number of MSB and single synaptic profiles per neuron increased about 8-fold from 7 to 27 DIV (Fig. 1B, bottom).

As a control, we also quantified puncta fitting the opposite profile: single PSD95 postsynaptic puncta in contact with multiple vGlut1 presynaptic puncta. Though synapses fitting this profile have been observed before [20], [21], we observed it for less than 1% of all contacts (Fig. 1B, top), suggesting that the MSB synapse profiles did not arise by chance. Quantification of all vGlut1 and PSD95 puncta, including lone puncta not part of synaptic profiles, showed that there was about the same number of vGlut1 and PSD95 puncta at 7 DIV but from 17 to 27 DIV, there were almost three vGlut1 puncta for every two PSD95 puncta (Fig. 1B, bottom). Because there are more vGlut1 puncta than PSD95 puncta, MSB synapse profiles are less likely to occur by chance and opposite profiles are more likely to occur by chance. Thus, our findings of an appreciable number of puncta forming MSB synapse profiles compared to a miniscule amount of opposite profiles, is the inverse of what would be expected had the profiles been formed by chance.

To obtain further evidence of MSB synapses in dissociated culture, we transfected neurons with cell-filling GFP to visualize dendritic spines then immunolabeled at 23 DIV with an antibody against SV2 to label boutons (Fig. 1C). Again, although the majority of SV2 puncta were in contact with single GFP-filled spines, we observed many in contact with multiple GFP-filled spines (Fig. 1C, arrows). These multiple contact spines generally were very close to each other and had heads angled towards or in parallel, which would be the likely configuration for an MSB-contacting spine pair. In these dissociated cultures, it is unlikely that spines from different dendrites contact MSBs because, as evidenced by our fluorescence and EM images, the dendrites do not grow in sufficiently close proximity for this to occur. Our laser scanning confocal microscopy (LSCM) observations point to the existence of MSBs in dissociated culture and further open the possibility that MSB synapse dynamics could be observed in live neurons by LSCM.

### Spatial properties of MSB-contacting spines

**Figure 2:**
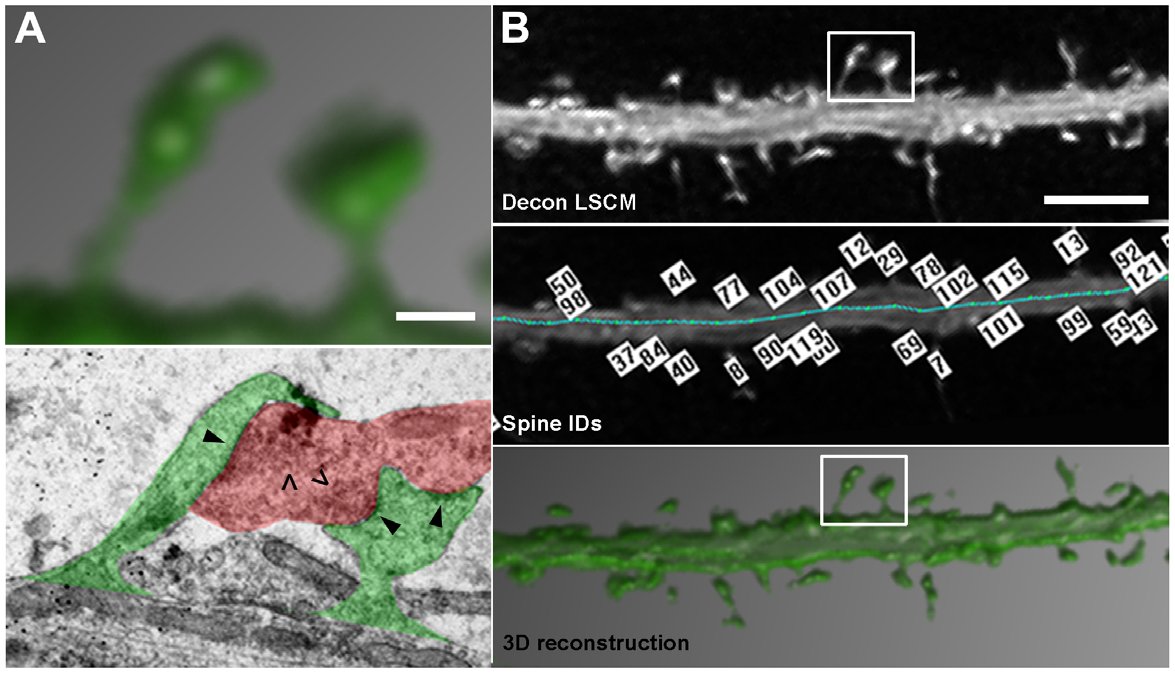
LSCM spine detection and CLEM verification of MSB-contacting spines. (A, top) 3D reconstruction of CLEM-verified MSB-contacting spines boxed in (B). Scale bar is 0.5 µm. (A, bottom) Single section EM of the spines boxed in (B, top). Bouton is shaded red. Spines are shaded green. Arrowheads point to postsynaptic densities. Carets point to presynaptic vesicles. (B, top) Deconvolved LSCM z-stack projection of a dendritic segment. A CLEM-verified MSB spine pair is boxed. Scale bar is 2 µm. (B, middle) NeuronStudio detection of spines and labeling with identification numbers. The blue line runs along the dendrite automatically traced by NeuronStudio. (B, bottom) 3D reconstruction of the same segment. See also Figure S1.

We used correlative light and electron microscopy (CLEM) of GFP-filled neurons to determine whether MSB synapses exist in dissociated neuron culture and to evaluate spatial properties of MSB-contacting spines with light microscopy. First, GFP-filled dendritic segments were imaged with LSCM then the neurons were processed for CLEM guided by grids on the coverslips that the neurons were grown on (see Materials and Methods). LSCM-imaged dendritic segments were relocated under the electron microscope and EM images were taken of spiny regions along the dendritic segments (Fig. S1). Individual spines could be identified in corresponding LSCM and EM images based on dendrite and spine morphology (Fig. 2A). To qualify as an MSB synapse, the bouton had to contain synaptic vesicles and both spines had to contain postsynaptic densities (Fig. 2A, bottom). In this manner, a total of 22 MSB-contacting spine pairs were identified in corresponding LSCM and EM images.

To evaluate spatial properties of the MSB spine pairs identified by CLEM, deconvolved LSCM z-stacks of the spines were imported into NeuronStudio for automated spine detection and analysis (Fig. 2B) [22], [23]. Spines were numbered (Fig. 2B, middle panel) and x-y-z coordinates were assigned to the center of each spine head. For controls, pairs of adjacent spines were randomly selected from along the same dendritic segments as the MSB spine pairs identified by CLEM. For each analysis, we used equal numbers of control and MSB spine pairs.

**Figure 3:**
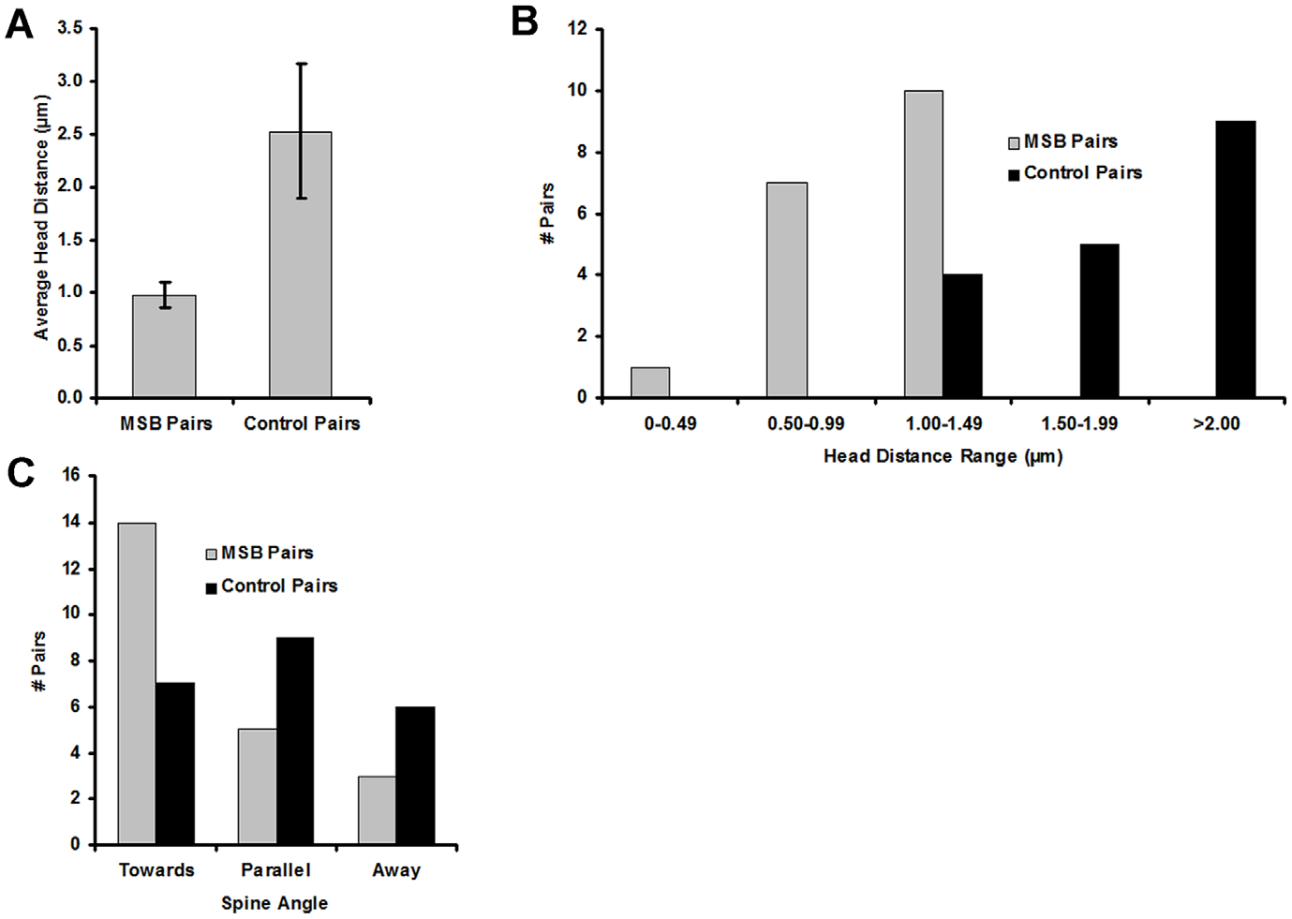
Spatial properties of MSB spine pairs. (A) Average spine head distances for 18 MSB-contacting and 18 control spine pairs. Distances were measured between the centers of mass of the spine heads. Error bars represent standard deviation. p = 4×10^−5^ by a one-tailed *t* test. (B) Spine head distance distribution of the MSB and control spine pairs. (C) Spine orientation distribution of 22 MSB and 22 control spine pairs. Spine orientation was manually determined as angled towards for heads nearer than bases, parallel for heads and bases equidistant, or angled away for heads further than bases. The 22 control pairs used for the orientation analysis are different because orientation was not manually discernable in the original 18 pairs. p = 0.006 by a χ^2^ test.

The distances between spine heads of control and MSB spine pairs were calculated from the x-y-z coordinates of the head centers. While it would likely be expected that MSB-contacting spines would be close together by virtue of their both contacting a single bouton, we nevertheless proceeded to determine the degree to which this was true compared to randomly selected control pairs of adjacent spines. If the MSB-contacting spines were significantly closer together than the control spines, this could be used as an MSB identification criterion. Indeed, the average spine head distance of the 18 MSB spine pairs analyzed was 0.98±0.12 µm (s.d.) while for the 18 control pairs it was 2.53±0.64 µm (s.d.) (Fig. 3A). The average distance for the MSB spine pairs was significantly less than that of the control pairs by a one-tailed *t* test (p = 4×10^−5^). All 18 MSB spine pairs analyzed for head distance had head distances less than 1.3 µm, while all but 3 of the 18 control pairs had head distances greater than this (Fig. 3B). The spine head distance distributions of MSB and control spine pairs were significantly different by a two-tailed Mann-Whitney U test (p = 5×10^−7^). The overall spine density was 0.75 spines per micrometer.

Spine orientation was determined manually by visual inspection. Again, while it would likely be expected that MSB-contacting spines would be oriented towards a single point by virtue of their both contacting a single bouton, we nevertheless proceeded to determine the degree to which this was true compared to the control spines. As with spine distance, if the MSB-contacting spines were significantly more likely to have a particular orientation, this could be used as an identification criterion. Indeed, the spines in the majority of the 22 MSB spine pairs analyzed were angled towards each other (14 of 22) while some were in parallel (5 of 22) and the least were angled away from each other (3 of 22). In the 22 control pairs analyzed, the spines were approximately equally likely to be in parallel (9 of 22), angled towards (7 of 22), or away (6 of 22) from each other (Fig. 3C). The distribution of the MSB spine pairs among orientation categories was significantly different from that of the control spine pairs by a χ^2^ test (p = 0.006).

### Spine stability at MSB synapses

**Figure 4:**
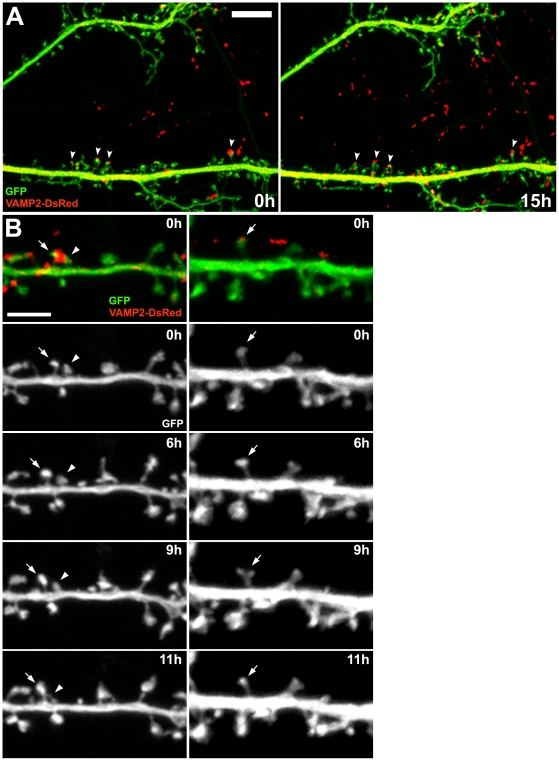
Long-term live LSCM of GFP-filled dendritic spines contacting VAMP2-DsRed-labeled boutons in dissociated cultured neurons. (A) Example showing many clear spinous synaptic contacts over 15 hours. Arrowheads point to clearly labeled spinous synapses that persisted over the entire duration. Scale bar is 10 µm. (B) Examples showing MSB and SSB synapses over 11 hours. Top panels (0 h) show live LSCM z-stack projections of GFP-filled dendrites (green) and VAMP2-DsRed-labeled boutons (red). In the bottom panels (0–11 h), the GFP detection channels are shown separated for clarity in discerning the dendritic spines. (Left panels) Arrow and arrowhead point to spines of an MSB spine pair. The spine with the arrow is persistent and stable while the one with the arrowhead is unstable and retracts. (Right panels) Arrow points to a persistent stable SSB-contacting spine. Scale bar is 5 µm. See also Movies S1, S2, and S3.

To evaluate the dynamics of MSB synapses, we used long-term time lapse live LSCM of pre- and postsynaptically labeled neurons in dissociated culture (Fig. 4A, Mov. S1). Transfected VAMP2-DsRed was used to label presynaptic boutons. Transfected cell-filling GFP was used to visualize the postsynaptic dendrite and its spines. The persistence of many clear VAMP2-DsRed-labeled presynaptic boutons in direct contact with GFP-filled dendritic spine heads over 15 or more hours (Fig. 4A, arrowheads) indicates that intact spinous synapses can be reliably observed and recorded with this system over long periods of time. The clarity and duration of this live microscopy system provides an excellent means of accurately observing both pre- and postsynaptic changes over time. Because VAMP2 is a functional presynaptic protein, it is possible that overexpression of the transfected VAMP2-DsRed could affect presynaptic neuron dynamics.

In the live LSCM images, MSB spine pairs were identified both by being in contact with single VAMP2-DsRed puncta and by spatial criteria derived from the CLEM observations; specifically, the spine heads had to be within 1.3 µm of each other and the spines had to be either angled towards each other or in parallel (Fig. 4B, left panels, arrow and arrowhead; Mov. S2). In this manner, we identified 4 MSB spine pairs in 3 independent live image series. For comparison, we also identified 7 SSB-contacting spines in 4 independent live image series, each in clear contact with a VAMP2-DsRed punctum (Fig. 4B, right panels, arrows; Mov. S3). The SSB-contacting spines were paired with nearby spines along the same dendrite. The live LSCM image series ranged from 15 to 42 hours in duration but, for matching across samples we used only 15 hours of each, beginning with the first time point at which the synapse of interest was clearly discernable.

**Figure 5:**
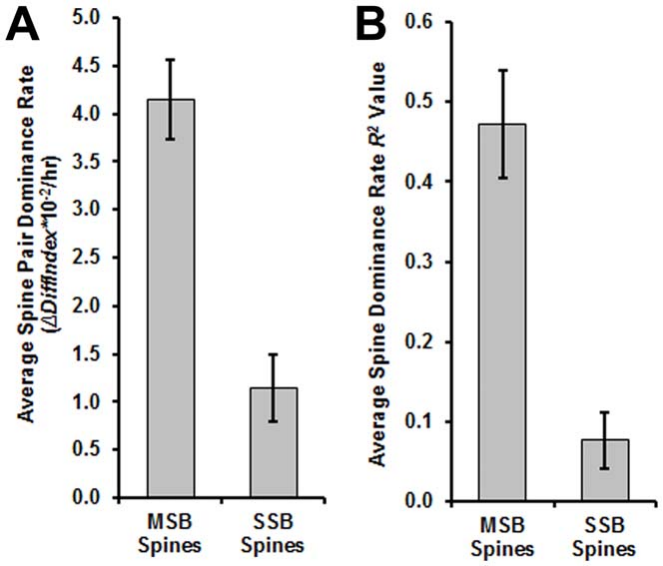
MSB-contacting spines are more dynamic than SSB-contacting spines. (A) Average dominance rates for MSB and SSB spine pairs. The dominance rate is a measure of the change in size difference over time between the dominant and nondominant spine. (B) Average *R^2^* values for the dominance rates of the MSB and SSB spine pairs. The *R^2^* values are a measure of the strength of the dominance trends. See the Methods section for further explanation of these calculations. For all graphs, error bars represent standard deviation. p = 0.001 for dominance rate (A) and p = 0.002 for *R^2^* value (B) by one-tailed *t* tests.

Spine stability for MSB and SSB spine pairs was evaluated by measuring the integrated density of each spine head over time then calculating a dominance rate for each spine pair based on changes in the integrated densities of the spine heads. Integrated density has been established as a measure of spine volume [24]. Thus, in short, the dominance rate is a measure of divergence in volume between spines of a pair over time. To measure spine stability, we calculated dominance rates of MSB and SSB spine pairs. for MSB spine pairs, the average dominance rate (0.042±0.004 Δ*DiffIndex*/hr, s.d.) was approximately 21-fold higher than that of the SSB spine pairs (0.002±0.007 Δ*DiffIndex*/hr, s.d.) (Fig. 5A). Also, the fact that the dominance rates were positive for each of the MSB spine pairs indicates that the larger spine was always the one that trended towards dominance. The difference between dominance rates was significant by a one-tailed *t* test (p = 0.001). Furthermore, the dominance rates of the MSB spine pairs had an approximately 6-fold higher average *R^2^* value (0.471±0.067 s.d.) than the SSB spine pairs (0.076±0.035 s.d.) (Fig. 5B), indicating that their dominance trend was strong. The difference between *R^2^* values was also significant by a one-tailed *t* test (p = 0.002).

**Figure 6:**
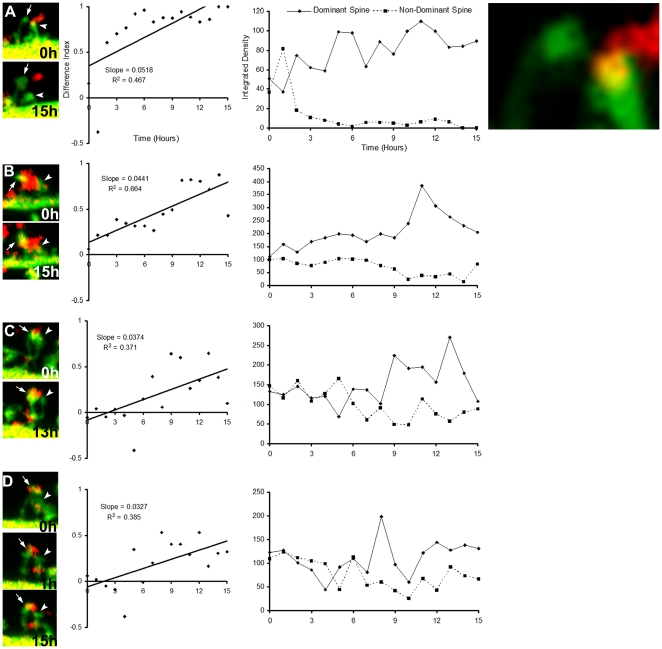
The dynamics exhibited by MSB-contacting spines is suggestive of competition between the spines. Left panels show the first (0 hour) and last (15 hour) time points from the time lapse LSCM image series of the MSB synapses analyzed. Arrows point to dominant spines. Arrowheads point to nondominant spines. In (D), the 1 hour time point is also shown as it more clearly shows the contact between the MSB and the nondominant spine. Due to presynaptic vesicle turnover, the VAMP2-DsRed bouton labeling is occasionally absent. Far right top panel is an enlargement of the MSB spine pair in (A) showing the rightmost tip of the spine of the left in abutting contact with the red bouton. Center panels are graphs of difference indices over time, calculated from the integrated densities of the spine heads (right panels). The slopes of the linear trend lines of the difference indices represent the dominance rates. Right panels are graphs of the integrated densities over time. See the Methods section for further explanation of these calculations.

**Figure 7:**
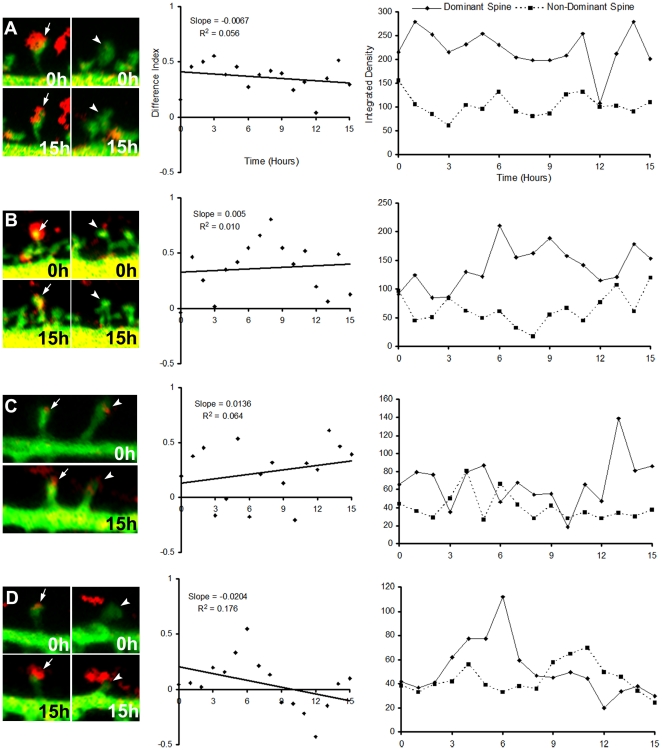
Randomly paired SSB spines do not exhibit dynamics suggestive of competition. Panel descriptions are the same as for Figure 7 except left panels show SSB synapses and their randomly paired spines.

The high dominance rates and *R^2^* values of the MSB spine pairs possibly reflect competition between the spines: coordinated enlargement of one spine and shrinking of the other (Fig. 6), sometimes ending in complete retraction (Fig. 6A). Although the SSB spine pairs also exhibited enlargement and shrinking, they did not do so in a coordinated manner or as rapidly and thus had low dominance rates and low *R^2^* values (Fig. 7). Finally, all the MSB spine pairs began with both spines having very similar integrated densities (Fig. 6, right panel). This suggests that the competition only begins after each spine has made a connection of equal strength with the MSB.

## Discussion

MSB synapses account for a significant percentage of excitatory synapses in many brain regions at basal levels and are formed following synaptogenic stimuli, suggesting an involvement in synaptic development or plasticity. This study is the first to use live imaging of pre- and postsynaptically labeled neurons to examine MSBs. Previously, live imaging of pre- and postsynaptically labeled neurons has been used to investigate synaptogenesis occuring on emerging dendritic protrusions and filopodia but these studies did not examine MSB synapses [21], [25]. Prior studies of MSB synapses employed static visualization techniques and so the dynamics and fate of individual MSB synapses over time have not been examined. The studies by Konur and Yuste (2004) and Ziv and Smith (1996) used FM loading to visualize boutons during live imaging of fluorescence-filled dendrites. However, the live imaging sessions in these studies were conducted on the order of minutes. In contrast, the ability of our transfected marker method to label many boutons continuously over long periods of time is what allowed us to prospectively identify MSBs, which are relatively rare structures. Therefore, it is quite likely that due to the short-term nature of their live imaging, these other authors did not encounter MSBs during their live imaging sessions.

Dissociated neuron cultures greatly facilitate live imaging of MSB synapses by allowing co-culture of two sets of neurons. By transfecting one set of neurons with a presynaptic marker and the other with a postsynaptic marker, it is possible to visualize both the pre- and postsynaptic components of an MSB synapse. With immunolabel of these cultures, we observed boutons that, by contacting multiple postsynaptic specializations, fit the expected profile of MSBs. We also observed immunolabeled boutons contacting multiple GFP-filled spines in cultures transfected with cell-filling GFP. These apparent MSBs were present to a degree comparable to that reported for MSB synapses *in vivo*. This evidence prompted us to investigate MSB synapses in live dissociated neuron cultures.

To characterize spatial properties of MSB-contacting spines, we imaged spiny dendritic segments in three dimensions with LSCM then used CLEM to definitively identify MSB-contacting spines. An analysis of the spines revealed unique properties of MSB-contacting spine pairs setting them apart from other spines. We then used long-term live LSCM of GFP-filled spines contacting VAMP2-DsRed-labeled boutons to evaluate spine dynamics and found that MSB-contacting spine dynamics possibly reflect competition for synaptic contact whereas SSB-contacting spine dynamics do not appear to resemble competitive behavior. Overall, our results suggest that MSB synapses may be dynamic intermediates in synaptic development.

### Possibilities for MSBs as dynamic structures in generation of neuronal connectivity

MSBs that contact multiple spines on the same dendrite rather than on different dendrites likely feature in mechanisms of synaptic reorganization. Following LTP induction in rat hippocampal slices, approximately two-thirds of newly grown MSB-contacting spines contact same- rather than different-dendrite MSBs [8]. In untreated rat hippocampal slices, however, only approximately 15% of MSBs are same-dendrite [4]. It is possible that same-dendrite MSBs formed through LTP [8], [15] may be less stable than different-dendrite MSBs. Indeed, a study using hippocampal slices found that, 2 hours after LTP induction by tetanic stimulation, there was no significant increase in the number of MSBs nor was there a significant change in total synapse number [26]. Thus, same- and different-dendrite MSBs may subserve different functions. Although the findings presented here were made with same-dendrite MSBs, the conclusions drawn may also apply to different-dendrite MSBs.

### Stability of MSB synapses

Our study presented here is the first to evaluate the stability of individual MSB synapses over time. Previous studies of synapse stability or motility did not utilize live imaging of simultaneous pre- and postsynaptic components over time periods as long as ours with images taken as frequently. However, some studies revealed that spine motility and synaptic contact are linked. First, it was shown by live imaging in mouse cerebellum slices that dendritic spines maintain motility while in synaptic contact [27]. This study only included live imaging of the spines themselves; the presynaptic contacts were confirmed *post hoc* with retrospective EM. When dendritic spines are not in synaptic contact, however, they exhibit decreased motility [28] as shown by simultaneous live imaging of dendritic spines and presynaptic boutons in cultured neurons. Finally, in mouse barrel cortex *in vivo* and in rat hippocampal slices, both stable and dynamic spines can be found along the same dendrite [29], [30] and these dynamics can be influenced by but do not depend on synaptic activity [30].

How MSBs participate in synaptic development or plasticity has yet to be demonstrated but some ideas have been proposed. One is that they provide a means of interneuronal propagation of LTP by retrograde signaling from the potentiated spine to the MSB, inducing potentiation of the other synapses of the MSB [6]. In this way, MSBs that contact spines of dendrites from different neurons could correlate neuronal populations not directly connected by synapses. Another idea, based on the EM finding that MSBs are more likely to contact young rather than old spines, is that MSBs resolve into separate synapses, lose contact with spines, or do both [15].

Our live LSCM data suggest that MSB-contacting spines compete for synaptic contact with the MSB. Possibly, one of the spines contacting the MSB receives less stimulation than the other and thus shrinks and retracts while the other spine grows larger. Indeed, an immunogold EM study of AMPA and NMDA receptors on MSB-contacting spine pairs found that the number of AMPA and NMDA immunogold particles was nearly five-fold greater on one of the two spines [31]. Such a difference in synapse strength could lead to strengthening of the stronger synapse and weakening or retraction of the other. This is consistent with the dominance rates being positive for each of the preselected larger spines of the pairs in our spine stability analysis. By losing contact with one of the spines while maintaining contact with the other, MSBs could provide a mechanism for synaptic reorganization without resulting in net synaptogenesis [17]. Finally, spine turnover and MSB formation both occur following sensory experience [10], [29], suggesting that these two processes are linked.

## Materials and Methods

### Animals used for neuron cultures

Pregnant female Sprague-Dawley rats were purchased from Charles River Laboratories (Wilmington, MA) and housed in the Center for Comparative Medicine and Surgery at the Mount Sinai School of Medicine which holds an Animal Welfare Assurance Number (A3111-01) signifying that it adheres to all National Institutes of Health guidelines for the care and treatment of laboratory animals. To obtain embryos for neuron culture preparation, pregnant rats were euthanized at E18.5 by CO_2_ asphyxiation and the embryos removed by caesarean section. All procedures were approved by the Mount Sinai School of Medicine Institutional Animal Care and Use Committee (IACUC) (protocol number 04-0548).

### Neuron culture preparation and immunocytochemistry

To prepare dissociated neuron cultures for CLEM analysis of GFP-filled spines, hippocampi were harvested from E18.5 rat embryos and dissociated. 3–4.5×10^6^ neurons were electroporated with 3 µg pEGFP-N1 plasmid DNA using the Rat Neuron Nucleofector Kit (Lonza Group Ltd., Basel, Switzerland) and plated on gridded glass bottom dishes (MatTek Corp., Ashland, MA) at approximately 1.7×10^5^ neurons/dish in MEM (Invitrogen, Carlsbad, CA) supplemented with L-glutamine. Before plating, the glass was treated for 12–24 hours with 1 M hydrochloric acid, washed 3×20 minutes with water, left to dry completely, treated for 8 hours with 1 mg/ml poly-L-lysine (Sigma-Aldrich, St. Louis, MO) in borate buffer, washed 3×10 minutes with water, then left to dry completely. Four hours after plating, the MEM was replaced with Neurobasal (Invitrogen) supplemented with B27 (Invitrogen) and L-glutamine. Neurons remained in this media at 37°C and 5% CO_2_ until fixation at 21 DIV with 4% glutaraldehyde in 0.1 M cacodylate buffer.

To prepare dissociated neuron cultures for immunocytochemistry, neurons were obtained as above and plated on 18 mm diameter coverglasses (Fisherbrand coverglass for growth, Thermo Fisher Scientific, Waltham, MA) then fixed with 4% paraformaldehyde at 7 to 27 DIV. Prior to plating, these coverglasses were treated with >65% pure nitric acid for 48–72 hours, washed 3×20 minutes with water, then sterilized and dried completely in an oven. Immunolabeling was carried out as described [32] using primary antibodies against vGlut1 (Millipore, Billerica, MA), PSD95 (Thermo Fisher Scientific), and SV2 (Developmental Studies Hybridoma Bank, University of Iowa, Iowa City, IA).

To prepare dissociated neuron cultures for long-term live laser scanning confocal microscopy (LSCM), neurons were again obtained as above and divided equally between two tubes, each containing 1.5–4.5×10^6^ neurons. The neurons in one tube were electroporated with 3 µg VAMP2-DsRed plasmid DNA (obtained from Dr. Kimberley McAllister, U.C. Davis) [33] and the other with 3 µg pEGFP-N1 as described above then plated in glass bottom dishes at a ratio of 1.5 VAMP2-DsRed to 1 GFP-transfected neuron at a total density of 4×10^6^ neurons/dish. This density and plating ratio maximized the number of axodendritic contacts between GFP-filled spines and VAMP2-DsRed-labeled boutons. The cultures were grown for 14 to 25 DIV before long-term live LSCM.

### LSCM

LSCM was performed using a Zeiss LSM 510 META (Carl Zeiss, Inc., Oberkochen, Germany) with a 63× oil objective (plan apochromat, numerical aperture 1.4). In addition, a 10× air objective was used to obtain low magnification images of the neurons in their locations on the gridded coverslips to facilitate neuron relocation for CLEM. The EGFP was excited by 488 nm wavelength argon laser light and its emission detected through a long pass 505 nm filter through a pinhole set to 1 Airy unit. For the 63× LSCM, z-stacks of 50 to 100 images were taken with an x-y pixel size of 0.14 µm^2^, an optical slice thickness of 0.7 µm and a z-step of 0.10, 0.14, 0.33, or 0.37 µm. The number of images in each z-stack depended on the thickness of the dendritic segment in the stack. Automated analysis of spine head distance was performed only with z-stacks taken with a 0.10 or 0.14 µm z-step. The scan speed was 3.2 µs/pixel with 4 line averaging. Z-stacks were deconvolved using AutoDeblur (version X1.4.1, MediaCybernetics, Bethesda, MD) prior to automated spine analysis in NeuronStudio.

For long-term live LSCM, the same 63× objective was used, the EGFP was excited by and detected through the same laser and filter, the x-y pixel size was 0.14 µm^2^, the optical slice thickness was 0.7 µm, the z-step was always 0.37 µm, and the scan speed was 1.6 µs/pixel with 2 line averaging. DsRed tagging VAMP2 was excited by 543 nm wavelength helium neon laser light and its emission detected through a band pass 585–615 nm filter. The GFP and DsRed were detected independently in separate channels. Z-stacks were taken every hour for 8 to 35 hours with a scan time of 3 to 10 minutes for each image depending on the size of the region in the z-stack. Prior to placing the dish on the microscope, the microscope chamber was equilibrated to 37°C and 5% CO_2_ for at least 1 hour. After focusing the objective on the dendritic segment to be recorded, the dish was left to equilibrate for at least 1 more hour to prevent vertical drift during the long-term live LSCM. Dishes were covered with a PTFE (polytetrafluoroethylene) membrane (DuPont, Wilmington, DE) sealed around the dish using a rubber band to prevent media evaporation while allowing gas exchange. This covering has been used previously to ensure long-term survival during live microscopy [34].

### Neuron processing for CLEM

After conducting LSCM of GFP-filled dendritic segments, synaptic contacts were verified by CLEM using methods previously described [35], [36], [37]. Briefly, the neurons in the dishes were treated with 1% osmium tetroxide plus 1.5% potassium ferricyanide in 0.1 M cacodylate buffer, dehydrated in an ascending ethanol series (50%, 60%, 70%), left in 3% uranyl acetate in 70% ethanol for 12 hours at 4°C, washed in 70% ethanol, then further dehydrated in an ascending ethanol series (80%, 90%, 100%). The neurons were infiltrated with a 1∶1 solution of resin (Embed 812 kit, Electron Microscopy Sciences) and 100% ethanol for 24 hours at room temperature. The resin-ethanol solution was then replaced with a thin layer of pure resin and neurons of interest were embedded and sectioned through at 70 nm. The grid on the coverslip transferred to the resin (Fig. S1C) and was used as a guide during sectioning. Serial sections were collected on Formvar-coated slot grids. Sections were contrasted with lead citrate and uranyl acetate and serial sections of the cell of interest were recorded on an Hitachi H-7000 (Hitachi, Ltd., Tokyo, Japan) transmission electron microscope at 12–40 k magnification and 75 kV voltage. High (63×) and low (10×) magnification LSCM images were used as maps for relocating regions of interest based on dendrite and spine morphology.

### Automated dendritic spine detection

Deconvolved LSCM z-stacks of GFP-filled dendritic segments containing both CLEM-verified MSB spine pairs and control pairs of randomly selected adjacent spines were loaded into the NeuronStudio program (available at http://research.mssm.edu/cnic/tools.html) for automated analysis [22], [23]. Only well-isolated, well-developed spiny dendritic segments not in contact with other dendrites, glial cells, or excessive axons were selected for analysis. Dendritic segments of interest were selected manually then traced and reconstructed in 3D for automated spine detection. Tracing errors and non-spinous entities misidentified as spines were eliminated manually. All spines were numbered for identification.

Spine parameters for both MSB and control spine pairs were imported into Excel (Microsoft Corp., Redmond, WA) for analysis. The distance between spine heads was measured as the distance between the centers of mass of the heads using the x-y-z coordinates determined by NeuronStudio. Spine orientation was determined manually by visual inspection of the reconstructed dendrite in 3D. For this analysis, the set of 22 control spines was different than that used for the other analyses because spine orientation had to be manually determined and was not clearly discernable in the original set. Spine with heads nearer than bases were classified as angled towards each other, spines with heads further than bases were classified as angled away from each other, and spines with equidistant heads and bases were classified as in parallel.

### Spine stability analysis

Live LSCM z-stack projections containing both MSB and SSB synapses were loaded into ImageJ (version 1.42q, freely downloadable from the ImageJ website, http://rsbweb.nih.gov/ij/) for processing and analysis. Brightness and contrast were adjusted separately for the VAMP2-DsRed and GFP detection channels and noise was reduced separately for each channel by automatic removal of outlier pixels, defined as single pixels with any brightness having only completely dark adjacent neighbors. To match the duration across each live LSCM series, only the first 15 hours of each series were included for analysis.

Spine stability was evaluated for MSB spine pairs compared to SSB spines paired with their nearest clearly observable neighbors. The volume of each spine head was measured as the integrated density of the GFP fluoresence of each spine head calculated by ImageJ. Integrated density is mean brightness multiplied by area. The area of each spine head was defined manually by tracing the contour of each spine head. To normalize the data, the dominant spine for each pair was preselected as the spine with the higher average integrated density over the 15 hour LSCM series. Then, a difference index was calculated for each time point as the integrated density of the dominant spine minus the nondominant spine divided by the sum of the two according to the formula:
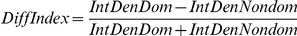
where *DiffIndex* is the difference index, *IntDen* is the integrated density, *Dom* is the dominant spine, and *Nondom* is the nondominant spine.

The difference indices were plotted over time from frame to frame using Excel and fit to linear trend lines. The slopes of the trend lines were taken as a measure of the degree of dominance, or the dominance rate. *R^2^* values were also calculated for the trend lines.

### Statistical analyses

One-tailed *t* tests were used to compare spine head distances and dominance rates for MSB-contacting versus control spines. A two-tailed Mann-Whitney U test was used for the spine head distance distribution. A χ^2^ test was used for the spine orientation distribution. Significance was set at an α level of 0.05 for all tests.

## Supporting Information

Figure S1 CLEM process for dissociated cultured neurons. Neuron of interest is shown boxed in (A) DIC showing neuron and grid, (B) fluoresence showing GFP-filled neuron, and (C) block face of neuron in grid. EM of boxed neuron is shown in (D). See also Figure 2.
(TIF)

Movie S1 Long-term time lapse live LSCM z-stack projection sequence of GFP-filled dendrites in contact with VAMP2-DsRed-labeled boutons in dissociated culture. Wider field of same neuron as in Figure 5A. Scale bar is 10 µm. See also Figure 5.
(AVI)

Movie S2 Long-term time lapse live LSCM z-stack projection sequence of an MSB synapse. Same GFP-filled dendritic segment in contact with VAMP2-DsRed-labeled boutons as in Figure 5B, left panel. Arrow and arrowhead point to spines of an MSB-contacting spine pair. The spine with the arrow is persistent and stable while the one with the arrowhead is unstable and retracts. Scale bar is 5 µm. See also Figure 5.
(AVI)

Movie S3 Long-term time lapse live LSCM z-stack projection sequence of SSB synapses. Same GFP-filled dendritic segment in contact with VAMP2-DsRed-labeled boutons as in Figure 5B, right panel. Arrows point to persistent stable SSB-contacting spines. Scale bar is 5 µm. See also Figure 5.
(AVI)
