# Supplementary figures and images for: Characterization of MSB Synapses in Dissociated Hippocampal Culture with Simultaneous Pre- and Postsynaptic Live Microscopy
Source: PLoS One. 2011 Oct 20;6(10):e26478. doi: 10.1371/journal.pone.0026478 (PMC3197663; doi:10.1371/journal.pone.0026478)

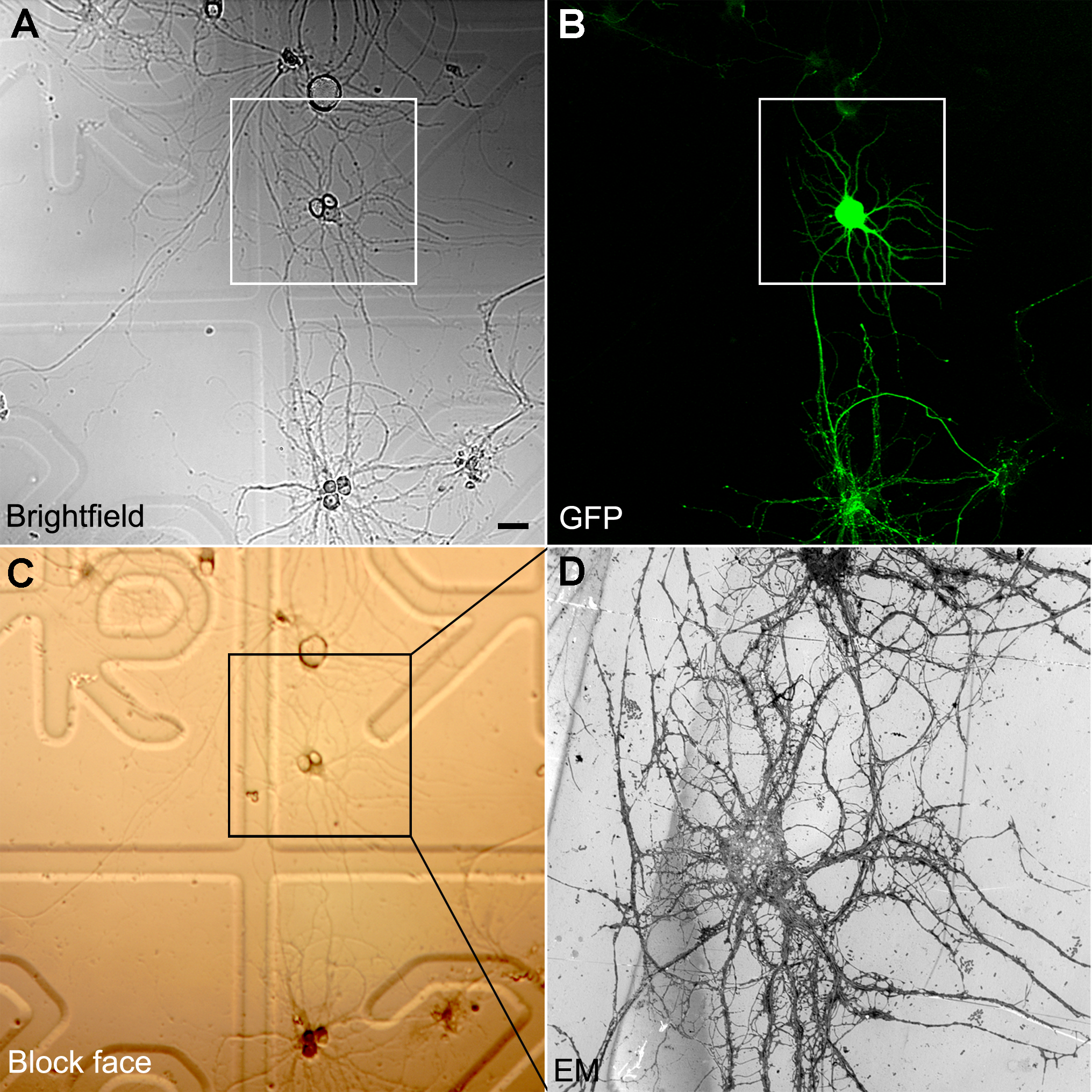

Supplement: Figure S1 — CLEM process for dissociated cultured neurons. Neuron of interest is shown boxed in (A) DIC showing neuron and grid, (B) fluoresence showing GFP-filled neuron, and (C) block face of neuron in grid. EM of boxed neuron is shown in (D). See also Figure 2. (TIF) [file pone.0026478.s001.tif]
